# Secondary left heart failure occurred during VA-ECMO assistance for severe residual pulmonary hypertension after pulmonary endarterectomy: a case report

**DOI:** 10.1186/s12871-021-01534-z

**Published:** 2021-12-18

**Authors:** Feng Long, Ming Luo, Zhen Qin, Bo Wang, Ronghua Zhou

**Affiliations:** Department of Anesthesiology, West China Hospital of Sichuan University, Chengdu, Sichuan 610041 PR China

**Keywords:** Heart failure, Extracorporeal membrane oxygenation, Cardiopulmonary bypass, Pulmonary endarterectomy, Chronic thromboembolic pulmonary hypertension

## Abstract

**Background:**

In patients of chronic thromboembolic pulmonary hypertension undergoing pulmonary endarterectomy, veno-arterial extracorporeal membrane oxygenation (VA-ECMO) provides full haemodynamic support. However, during a rescue treatment of VA-ECMO for patients with difficulty weaning from cardiopulmonary bypass, a significantly increase left ventricular afterload through retrograde infusion of arterialized blood into the descending aorta may occur.

**Case presentation:**

We report a 70-year-old man who suffered severe residual pulmonary hypertension following pulmonary endarterectomy for chronic thromboembolic pulmonary hypertension. Preoperative echocardiogram showed a dilated and poorly functioning right ventricle, as well as a small left heart with normal function (TAPES9.6 mm, LVEF64%, average E/E′11.94, lateral E′12.1 cm/s, tricuspid regurgitation velocity 2.5 m/s), while postoperative echocardiography revealed a significant decrease of whole ventricular function on postoperative day 1(TAPES4mm, LVEF28%, average E/E′15, lateral E′6.7 cm/s, tricuspid regurgitation velocity 4.1 m/s), indicating the patient developed severe secondary left ventricular dysfunction on the basis of right ventricular dysfunction, during VA-ECMO support. Then comprehensive measures were adopted, such as down-regulating VA-ECMO flow rate, adjusting respiratory parameters, using vasoactive drugs, as well as prostacyclin. Eventually, the pulmonary hypertension decreased to moderate degree, and the heart function improved gradually.

**Conclusions:**

In the face of severe residual pulmonary hypertension and sencondary left ventricular dysfunction associated with VA-ECMO, comprehensive measures described above may facilitate recovery. ECMO flow titration to maintain relatively low flow rate is very important to not only maintain systemic perfusion, but also reduce left ventricular afterload and ensure pulsatile perfusion of pulmonary artery.

## Background

In patients of chronic thromboembolic pulmonary hypertension (CTEPH), there can be an impaired right and left ventricular function [1]. While improved lung perfusion and the reduction of right ventricular pressure overload, which performed as reduction in right ventricular size, improvement in the left ventricular diastolic and systolic function, as well as the cardiac index, can be found after pulmonary endarterectomy (PTE), residual pulmonary hypertension (PH) is often present, with the rate up to 51% [1, 2]. At this condition, veno-arterial extracorporeal membrane oxygenation (VA-ECMO) provide an rescue treatment for patients with difficulty weaning from cardiopulmonary bypass. However, it is increasingly recognized that VA-ECMO, especially peripheral VA-ECMO, may significantly increase left ventricular (LV) afterload through retrograde infusion of arterialized blood into the descending aorta, despite its efficacy of providing full haemodynamic support. As a consequence of this inherent limitation of VA-ECMO, the myocardium is overloaded and impaired, which significantly limits cardiac recovery, and negatively impacts on long-term prognosis [3].

## Case presentation

**Fig. 1:**
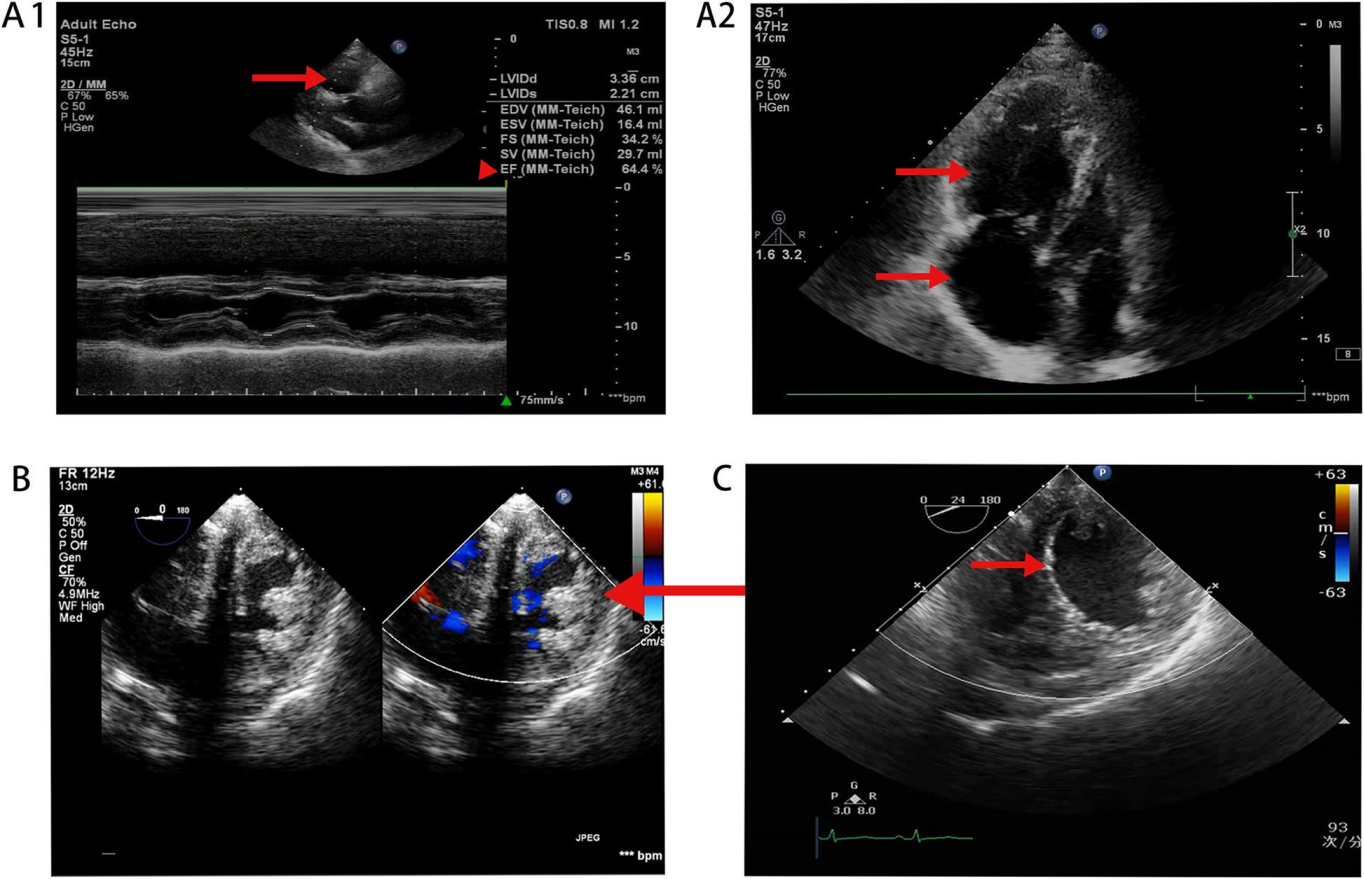
Perioperative echocardiography. Figure 1-A1, A2: Preoperative echocardiography revealed the greatly enlarged right heart with decreased systolic function, and small left heart with normal systolic function (LV34 LA21 RV36 RA49 EF 64.4%). B On POD 1, right heart still dilated significantly with high ventricular pressure, while left heart shrunk obviously and presented like the form of D (as the arrow points to), which indicate left ventricular dysfunction. C On POD 7, echocardiography showed a normal sized heart with satisfying ventricular systolic function. As is shown, right heart had retracted apparently and left heart had recovered to the size of O (as the arrow points to) with improved ventricular function

A 70-year-old male, diagnosed with CTEPH, was hospitalized for PEA. He had post-exercise exhaustion and shortness of breath for almost 2 years, and experienced a sudden dizziness with visual rotation and syncope. Despite the medical therapy, the clinical symptoms gradually worsened. Echocardiogram showed a dilated and poorly functioning right ventricle, as well as a small left heart with normal systolic and diastolic function (Fig. 1-A1, A2)(Tricuspid annular plane systolic excusion (TAPSE) 9.6 mm, left ventricular ejection fraction (LVEF) 64%, average E/E′11.94, lateral E′12.1 cm/s,tricuspid regurgitation (TR) velocity 2.5 m/s). Ventilation/perfusion scan and computed tomography angiogram confirmed the presence of CTEPH at the sub-segmental levels.

After the patient was induced, the initial pulmonary arterial pressure (PAP) was 93/46(63) mmHg, and preoperative right radial artery blood pressure (ABP) was 120/88 mmHg. Near infrared spectroscopy (NIRS) monitoring presented the basic cerebral regional oxygen saturation (rSO_2_) range from 60 to 65%. After median sternotomy, the ascending aorta and both vena cava were cannulated regularly, and CPB was initiated. After the initiation of CPB, the mean PAP (mPAP) decreased to the range from17 to 31 mmHg. Aortic cross-clamping and blood cardioplegia were administered during pulmonary arteries dissociation. Then, the removal of thickened arterial intima and old organized thrombi was completed under deep hypothermic (22 °C) low flow (DHLF) and deep hypothermic circulatory arrest (DHCA). NIRS fluctuates from 55 to 60% during DHLF and DHCA. After the surgical procedures, full flow was restored, heart rebeated, and the patient was gradually rewarmed to normal. Norepinephrine (0.05μg/kg.min), epinephrine (0.02μg/kg.min), and isoproterenol (0.02μg/kg.min) were used to maintain stable hemodynamics. Transesophageal echocardiogram (TEE) showed good systolic and diastolic heart function.

**Fig. 2:**
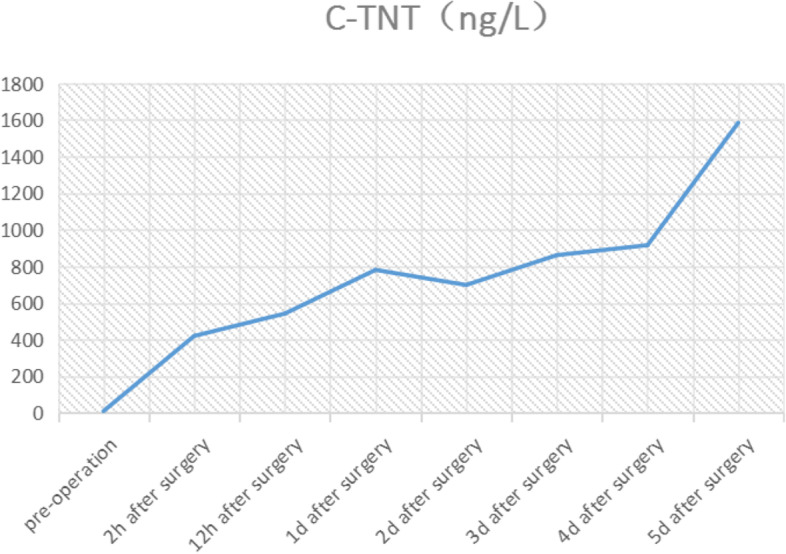
The postoperative troponin-T(C-TNT) increased gradually in the first five days under ECMO

However, during the first attempt to discontinue CPB, the PAP increased rapidly to 63/35(43) mmHg, with unstable hemodynamic (ABP 70/40 mmHg), and TEE suggested right heart insufficiency, accompanied with normal left ventricular function. Then, CPB was resumed immediately. After assisting for another 45 min and increasing the dosage of vasoactive drugs (norepinephrine 0.06μg/kg.min, epinephrine 0.05μg/kg.min, isoproterenol 0.05μg/kg.min), the separation failed again, just the same as the first time. Considering that severe residual PH combined with right heart dysfunction are the main reasons for the failure of CPB weaning, ECMO was adopted. Consequently, 19F arterial cannula and 22F venous cannula were inserted into the right femoral vessels, and femoral VA-ECMO was initiated with a flow of 3.0 ~ 3.5 L/min. On arrival at the intensive care unit, ECMO flow rate was 3.5 L/min, and severe PH (PAP 56/25 mmHg) persisted with low ABP (70/55 mmHg). Echocardiography revealed significant decrease of biventricular function (Fig. 1B)(TAPES4mm, LVEF28%, average E/E′15, lateral E′6.7 cm/s, TR velocity 4.1 m/s) on postoperative day (POD) 1, indicating the patient developed severe secondary left ventricular dysfunction on the basis of right ventricular dysfunction, during ECMO support. The postoperative troponin-T(C-TNT) increased gradually (Fig. 2), which suggested the myocardial injuries occurred. Hence, comprehensive measures were adapted: ECMO flow rate was reduced from 3.5 L/min to 2.5 L/min to decrease left ventricular afterload while maintaining systemic perfusion, norepinephrine (0.1μg/kg.min), epinephrine (0.06μg/kg.min) and isoproterenol (0.06μg/kg.min) were increased to ensure coronary perfusion, inhaled nitric oxide and treprostinil were administered to relieve PH. Besides, tidal volume was reduced to 6 ml/kg, and PEEP (5 cmH_2_O) was initiated to achieve lung protective ventilation. Oxygen flow rate was increased from 2 L/min to 6 L/min, with oxygen concentration raised to 47% for myocardial oxygen supply improvement. Moreover, atrial septostomy was carried out to decompress the right ventricle, so as to reduce the PAP. Consequently, mPAP decreased to 10 mmHg gradually, and the cardiac function improved slowly.

On POD 7, echocardiography revealed a normal sized heart with acceptable ventricular function (Fig. 1C). In this condition, ECMO was discontinued successfully, with satisfactory BP (90 ~ 120/50 ~ 80 mmHg), and normal PAP (mPAP 10-12 mmHg). The postoperative echocardiography data is shown in Table 1.

**Table 1:** Important data of postoperative echocardiography

	POD1	POD2	POD3	POD4	POD5	POD6	POD7 (before separation)	POD7 (after separation)
LVD (mm)	42	40	38	36	33	35	36	41
RVD (mm)	20	21	24	23	22	22	23	25
LVEF(%)	28	40	47	41	45	58	63	67
TAPSE (mm)	4	5	8	10	13	15	15	16
VTI	3	4.5	6	7.55	9	11	11.5	13.2

*POD* Postoperative day, *LVD* Left ventricular diameter, *RVD* Right ventricular diameter, *LVEF* Left ventricular ejection fraction, *TAPSE* Tricuspid annular plane systolic excusion, *VTI* Velocity time integral

The postoperative course was complicated by unexpected brain ischemia and luminal infarction, which required tracheostomy on POD 13. Finally, the patient’s family decided to transfer to local hospital for treatment On POD 16.

## Discussion and conclusions

Currently, the definition of residual PH after PEA has been updated by the 6th WSPH Task Force. Residual PH could be diagnosed when the mPAP is more than 20 mmHg, associated with pulmonary vascular resistance ⩾3 Wood Units postoperatively [4]. In this case, during the two attempts to separate from CPB, residual PH after PEA could be diagnosed. Postoperative severe residual PH and right heart dysfunction were considered to cause the difficulty weaning off CPB. For patients experiencing difficult separation from CPB, ECMO assistance is an effective choice.

Femoral peripheral VA-ECMO provides full haemodynamic support for patients but increases the afterload to the LV, attributable to retrograde aortic perfusion [5]. It’s reported that left ventricular overload at any time during VA-ECMO may occur in up to 70% of patients [6]. This could jeopardize myocardium and negatively affect survival. It’s very important to note that, secondary LV dysfunction occurred on the basis of right ventricular dysfunction during ECMO assistance in this case. The reasons for it can be summarized as follows: First, high flow rate of femoral VA-ECMO retrograde perfusion increases the left ventricular afterload. Second, due to chronically underfilled left ventricle, the untrained left ventricle cannot handle this increased volume load immediately after PEA, which can lead to temporary left heart failure and pulmonary edema. Moreover, the long period of CPB and DHCA may lead to myocardial ischemia reperfusion injury, eventually to heart insufficiency.

For LV dysfunction, it is well known that the insufficiency of the LV as a pump are manifested as systolic or diastolic dysfunction. The most frequent assessment of LV function based on echocardiography is the LVEF value, and patients LVEF values less than 55% usually have LV systolic dysfunction [7]. However, clinicians may pay more attention to LVEF for systolic function in perioperative echocardiographic evaluation sometimes, overlooking LV diastolic dysfunction (DDf). New guidelines for diagnosis of LV DDf based on transthoracic echocardiography have been simplified and focus on the increase in left atrial pressure (LAP) rather than grading diastolic dysfunction. In patients with normal LVEF, more than half of the following should be positive to determine the presence of DDf: average E/E′ > 14, lateral E′ < 10 cm/s, tricuspid regurgitation velocity > 2.8 m/s, and LA volume index > 34 ml/m2. In patients with depressed LVEF and with normal LVEF but myocardial disease, the mitral inflow ratio and the criteria above are used for evaluating LAP and grading DDf [8].

For the managment of LV dysfunction, we adjusted the femoral VA-ECMO flow to reduce left ventricular afterload, used vasoactive drugs to maintain blood pressure, for systolic ABP must be within 10–20% of the baseline value and pulse pressure (PP) should be kept below the diastolic ABP during intraoperative ABP control. And the “Rule of 70s” is useful for controlling ABP: age > 70, diastolic ABP > 70, Heart rate = 70, and PP < 70 [8]. Besides, respiratory parameters were adjusted to improve myocardial oxygen supply. In addition to the measures we took, the combination of VA-ECMO with an intra-aortic balloon pump (IABP) has long been advocated for LV unloading, which might help to create pulsatile flow and afterload reduction, reduce capillary wedge pressure, and avoid pulmonary oedema. However, its routine use during VA-ECMO is not unequivocally agreed upon for some conflicting clinical data. And the IABP may also negatively impact on the spinal cord and cerebral blood flow during VA ECMO, especially when the native cardiac function is severely impaired [3]. What’s more, the Impella, a trans-aortic LV assist device designed as a catheter-based, micro-axial impeller pump, can provide continuous blood flow from the LV into the ascending aorta, yielding very powerful LV unloading effects. A survival benefit and improved bridging to recovery when combining the Impella with VA ECMO were reported [3, 6]. For those diagnosed with DDf after surgery, continued and careful monitoring should be managed to prevent hypoxemia and atrial fibrillation, which are most common complications postoperatively. Maintenance of nitroglycerin at a low dose (25 μg/min) is useful for preventing heart failure by avoiding its’ risk factors, consisting of shivering, anemia, hypoxia, electrolytic imbalance, deterioration in diastolic dysfunction, myocardial ischemia, hypovolemia or hypervolemia, tachycardia, rhythms other than sinus, postoperative sympathetic stimulation and postoperative hypertensive crisis. In circulatory failure related to DDf, milrinone and levosimendanat are helpful. Milrinonea has inotropic, vasodilatory, and lusitropic (particularly in a heart failure state) effects with minimal chronotropy, by increasing calcium ion uptake in the sarcoplasmic reticulum. Levosimendanat sensitizes the contractile elements to calcium and has positive inotropic effects, modulating the interaction between troponin and calcium. It also has vasodilatory effects by opening ATP-sensitive potassium channels and improving both systolic and diastolic functions [8].

As to the ECMO mode chosen after PEA, veno-venous ECMO (VV-ECMO) is a good option for patients with only respiratory failure but preserved heart function, while VA-ECMO is crucial to heart failure cases with or without hypoxemia [9]. Given that our patient developed severe residual PH combined with right heart dysfunction, VA-ECMO was preferred. The choice between peripheral and central VA-ECMO should be considered in the context of the patient’s condition and the ECMO center’s experience. Harlequin syndrome is a known complication of peripheral VA-ECMO, where the upper part of the body is poorly oxygenated. It is essential to monitor the right radial artery blood gas to identify and prevent hypoperfusion of upper body and heart. In this case, increased vasoactive drugs for ensuring coronary perfusion, raised oxygen flow rate and oxygen concentration for myocardial oxygen supply improvement were expected to prevent Harlequin syndrome. We choose a peripheral cannulation strategy, for it can be easily done and reduce the risk of infection.

In conclusion, comprehensive measures such as down-regulating ECMO flow rate, adjusting respiratory parameters, using vasoactive drugs, as well as atrial septostomy may facilitate recovery, in the face of severe residual PH and sencondary heart dysfunction associated with ECMO. ECMO flow titration to maintain relatively low flow rate is very important to not only maintain systemic perfusion, but also reduce left ventricular afterload and ensure pulsatile perfusion of pulmonary artery.

## Data Availability

All data generated or analysed during our study are included in this published article and its supplementary information files.

## Abbreviations

CTEPH: Chronic thromboembolic pulmonary hypertension
PH: Pulmonary hypertension
PEA: Pulmonary endarterectomy
CPB: Cardiopulmonary bypass
LV: Left ventricle
ECMO: Extracorporeal membrane oxygenation
VA-ECMO: Veno-arterial extracorporeal membrane oxygenation
PAP: Pulmonary arterial pressure
mPAP: Mean PAP
POD: Postoperative day
DDf: Diastolic dysfunction
